# Supplementary material for: A Novel Predictive Model of Pathological Lymph Node Metastasis Constructed with Preoperative Independent Predictors in Patients with Renal Cell Carcinoma
Source: J Clin Med. 2023 Jan 5;12(2):441. doi: 10.3390/jcm12020441 (PMC9866659; doi:10.3390/jcm12020441)
Supplement: Supplementary file 1 [file jcm-12-00441-s001.zip › TableS1.pdf]

Table S1 Univariable logistic regression analysis of predictors of lymph node metastasis

	OR	95%CI	P-value
**Gender (Female vs Male)**	1.65	0.87-3.16	0.13
**Age**	0.96	0.94-0.99	<0.001
**ECOG PS (1 vs 0)**	2.12	1.09-4.11	0.03
**Complaint**			
Medical examination	-	-	Ref.
Lumbago	3.01	1.3-6.98	0.01
Hematuria	1.7	0.69-4.17	0.25
Others	4.41	1.69-11.5	<0.001
**Symptoms**			
None	-	-	Ref.
Local symptoms	2.55	1.24-5.22	0.01
Systemic symptoms	4.59	1.76-11.94	<0.001
**History of tumor (Yes vs No)**	2.59	0.51-13.24	0.25
**History of smoking and drinking (Yes vs No)**	0.78	0.38-1.61	0.5
**History of abdominal surgery (Yes vs No)**	1.06	0.43-2.6	0.9
**aCCI** (Age-adjusted Charlson comorbidity index)			
0	-	-	Ref.
1	0	0-Inf	0.99
2	0	0-Inf	0.99
3	0	0-1.18577408426107e^{304}	0.99
4	0.04	0-1.95148571749518e^{180}	0.99
5	0.21	0-2.03676787983067e^{81}	0.99
6	0.43	0-2.25477210041344e^{24}	0.98
**aCCI grade**			
Low (0-1)	-	-	Ref.
Middle (2-3)	0.75	0.35-1.63	0.47
High (≥4)	1.33	0.7-2.54	0.39
**Tumor side (Right vs Left)**	0.57	0.3-1.09	0.09
**Tumor size**	1	0.99-1.01	0.71
**Tumor size grade**			
≤7	-	-	Ref.
>7 and ≤10	1.33	0.77-2.3	0.3
> 10	1.42	0.75-2.68	0.28
**Exophytic or endophytic property**			
**50% or more exophytic**	-	-	Ref.
**Less than 50% exophytic**	0.93	0.41-2.12	0.87
**Entirely endophytic**	3.03	0.82-11.26	0.1
**Renal collecting system under pressure (Yes vs No)**	2.96	0.84-10.4	0.09

**Renal hilum invasion (Yes vs No)**	1.91	0.94-3.91	0.07
**Tumor morphology (Irregular vs Round)**	4.64	2.1-10.24	<0.001
**Pseudocapsule (Yes vs No)**	0.14	0.07-0.3	<0.001
**Tumor necrosis (Yes vs No)**	2.8	1.02-7.65	0.04
**Venous tumor thrombus**			
No	-	-	Ref.
Renal vein	1.68	0.71-3.97	0.24
Vena cava	0	0-Inf	0.99
**Lymph node status by pre-operative imaging**			
Negative	-	-	Ref.
Enlarged and increased	3.31	1.22-9.01	0.02
Metastasis	70.83	22.22-225.8	<0.001
**Lymph node size**	1.23	1.16-1.31	<0.001
**Lymph node size category by pre-operative imaging**			
≤10	-	-	Ref.
10-20	42.56	13.69-132.27	<0.001
> 20	0.99	0.43-2.3	0.98
**Lymph node fusion (Yes vs No)**	67	8.65-518.88	<0.001
**Distant metastasis (cM1 vs cM0)**	3.59	1.53-8.39	<0.001
**Neutrophil**	1.06	0.88-1.26	0.56
**Neutrophil category (Abnormal vs Normal)**	1.11	0.43-2.86	0.83
**Lymphocyte**	0.81	0.43-1.54	0.52
**Lymphocyte category (Abnormal vs Normal)**	0.89	0.37-2.15	0.8
**NLR**	1.03	0.88-1.2	0.72
**Hemoglobin**	0.99	0.98-1	0.21
**Anemia (Yes vs No)**	1.35	0.72-2.53	0.36
**Platelet**	1	1-1	0.23
**Abnormal platelet (Abnormal vs Normal)**	0.8	0.35-1.84	0.6
**Fibrinogen**	1.08	0.93-1.25	0.3
**Abnormal fibrinogen (Abnormal vs Normal)**	1.7	0.88-3.28	0.12
**D-dimer**	1.17	0.96-1.43	0.12
**D-dimer (High vs Normal)**	2.49	1.28-4.84	0.01
**Clotting time (Prolonged vs Normal)**	1.29	0.66-2.52	0.46
**Albumin**	0.95	0.89-1.02	0.14
**Albumin (Low vs Normal)**	1.28	0.61-2.67	0.51
**Globulin**	1.02	0.98-1.06	0.35
**Globulin (High vs Normal)**	1.75	0.91-3.39	0.09

**AGR**	0.53	0.22-1.28	0.16
**AGR (<1.5 vs Normal)**	1.6	0.73-3.51	0.24
**ALP**	1	0.99-1.01	0.71
**ALP (High vs Normal)**	0.36	0.08-1.66	0.19
**LDH**	1	1-1	0.46
**LDH (High vs Normal)**	3.19	1.48-6.87	<0.001
**Calcium**	3.11	0.6-16	0.17
**Calcium (High vs Normal)**	1.6	0.65-3.91	0.3
**Creatinine**	1	0.99-1.01	0.86
**Creatine (High vs Normal)**	1.09	0.46-2.55	0.85
**eGFR**	1.01	1-1.02	0.23
**Urine HBC**	1	1-1	0.2
**Urine occult blood (Yes vs No)**	3.09	1.61-5.93	<0.001
**Urine WBC**	1	1-1	0.21
**Urinary tract infection (Yes vs No)**	2.37	1.23-4.57	0.01
**Urine protein**	3.53	1.74-7.18	<0.001
**Clinical T category**			
cT1	-	-	Ref.
cT2	7.21	3.21-16.21	<0.001
cT3	1.56	0.69-3.52	0.28
cT4	0.38	0.17-0.85	0.02
**cT1-2 vs cT3-4**	4.13	2.5-6.82	<0.001

*Starting from “tumor size” to “venous tumor thrombus”, all the factors were collected from pre-operative imaging.
