# Supplementary figures and images for: A Novel Predictive Model of Pathological Lymph Node Metastasis Constructed with Preoperative Independent Predictors in Patients with Renal Cell Carcinoma
Source: J Clin Med. 2023 Jan 5;12(2):441. doi: 10.3390/jcm12020441 (PMC9866659; doi:10.3390/jcm12020441)

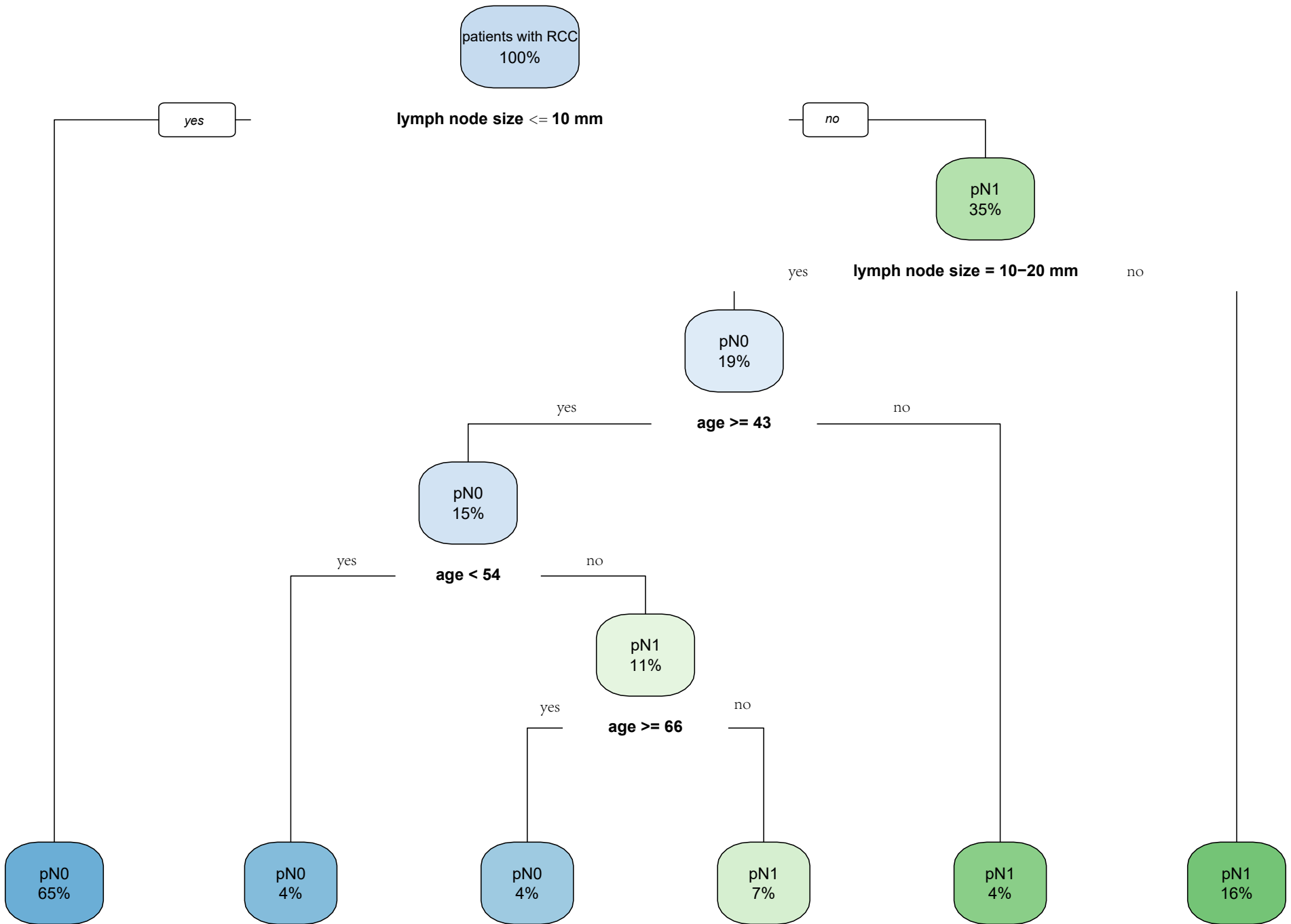

Supplement: Supplementary file 1 [file jcm-12-00441-s001.zip › FigureS1-Decision tree.pdf]

a

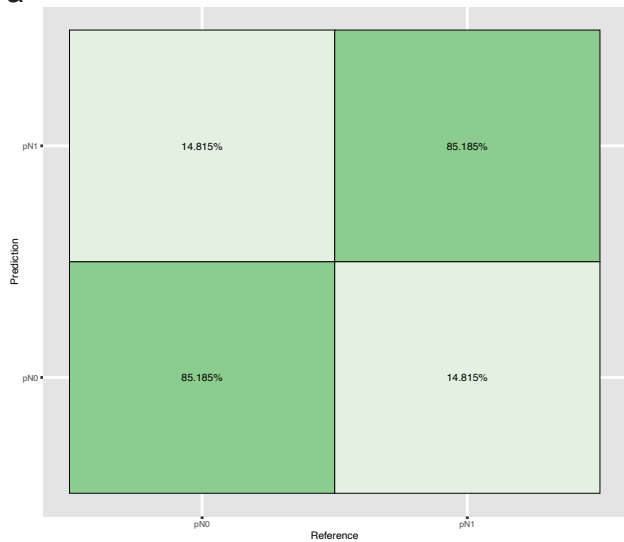

b

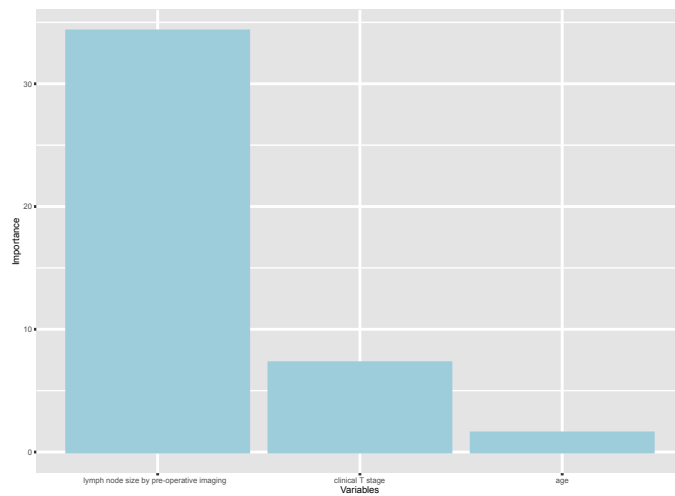

c

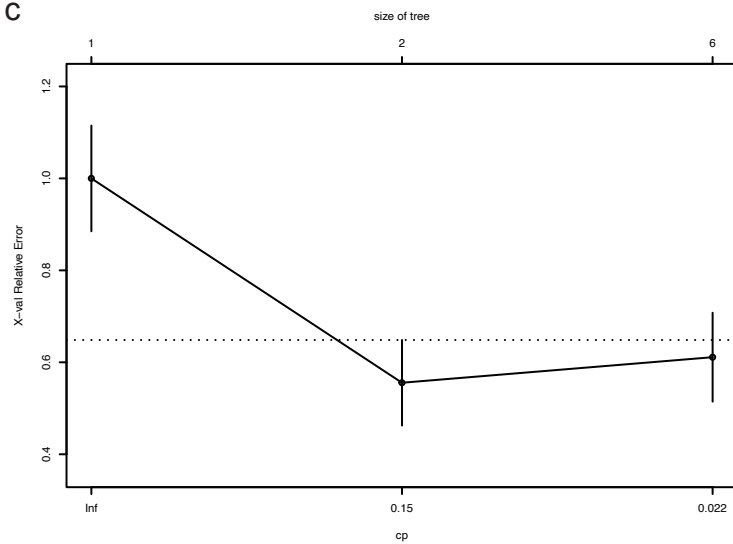

Supplement: Supplementary file 1 [file jcm-12-00441-s001.zip › FigureS2-The production process of the tree.pdf]
